# Genome Variability and Gene Content in Chordopoxviruses: Dependence on Microsatellites

**DOI:** 10.3390/v7042126

**Published:** 2015-04-22

**Authors:** Eneida L. Hatcher, Chunlin Wang, Elliot J. Lefkowitz

**Affiliations:** 1 Department of Microbiology, University of Alabama at Birmingham, BBRB 276/11, 845 19th St S, Birmingham, AL 35222, USA; E-Mail: eneidao@uab.edu; 2 Stanford Genome Technology Center, Stanford University, 855 California Ave, Palo Alto, CA 94304, USA; E-Mail: wangcl@stanford.edu

**Keywords:** poxviruses, genomic evolution, genome variability, early stop mutations, microsatellites

## Abstract

To investigate gene loss in poxviruses belonging to the *Chordopoxvirinae* subfamily, we assessed the gene content of representative members of the subfamily, and determined whether individual genes present in each genome were intact, truncated, or fragmented. When nonintact genes were identified, the early stop mutations (ESMs) leading to gene truncation or fragmentation were analyzed. Of all the ESMs present in these poxvirus genomes, over 65% co-localized with microsatellites—simple sequence nucleotide repeats. On average, microsatellites comprise 24% of the nucleotide sequence of these poxvirus genomes. These simple repeats have been shown to exhibit high rates of variation, and represent a target for poxvirus protein variation, gene truncation, and reductive evolution.

## 1. Introduction

The *Poxviridae* family consists of viruses with a wide host range and potential for causing disease. The family is divided into subfamilies based on host range, consisting of the *Entomopoxvirinae* which infect insects, and the *Chordopoxvirinae* (ChPV) which infect vertebrates [1]. The chordopoxviruses are further divided into several genera, including the *Orthopoxvirus* genus, which includes the most well-known and the best characterized poxviruses, variola virus and vaccinia virus. Smallpox disease is caused by the viruses belonging to the *Variola virus* (VARV) species, and is well known both for its severity and its eradication through a worldwide vaccination effort. The vaccine used was based on viruses belonging to the species *Vaccinia virus* (VACV), which at some point replaced cowpox virus that was originally used in Jenner’s smallpox vaccine, although the natural host of vaccinia viruses is unknown [2]. In addition to the *Orthopoxvirus* genus, chordopoxviruses are categorized into nine other genera, namely *Avipoxvirus*, *Capripoxvirus*, *Cervidpoxvirus*, *Crocodylidpoxvirus*, *Leporipoxvirus*, *Molluscipoxvirus*, *Parapoxvirus*, *Suipoxvirus*, and *Yatapoxvirus*, as well as the unassigned species *Squirrelpox virus*, and the yet to be classified, yoka poxvirus (YKPV) [1,3].

Poxvirus genomes consist of linear double-stranded DNA of 133,000–360,000 bp bounded by inverted terminal repeats [4]. Poxviruses code for 133–328 genes, and at least 90 gene families exhibit significant homology across the ChPV subfamily [4]. Viruses assigned to the same genus exhibit higher degrees of gene conservation. For example, all orthopoxviruses share a core set of approximately 174 genes, however, the remaining genes outside of this core set are always present among the set of 214 genes present in cowpox viruses, in addition to the other orthopoxviruses that may also contain the gene [4]. Poxviruses are unique among the DNA viruses because DNA replication occurs in the cytoplasm as opposed to the nucleus [5,6]. The large coding capacity of their genomes is one of the ways poxviruses support replication in the absence of the cellular DNA replication machinery, by coding for many of their own transcription and replication proteins. Most poxvirus genomes share similar genetic organization, with genes involved in essential functions such as transcription, DNA replication, and virion assembly located in a highly conserved central region [7]. Genes involved in immunomodulation and host range determination tend to cluster at the ends of the genomes, which are much more variable in terms of sequence homology and gene content.

Viruses within the species *Cowpox virus* (CPXV) have the largest genomes in comparison to other orthopoxviruses, and are believed to be most genetically similar to the ancestral orthopoxvirus in terms of gene content [4,8]. Several species including *Cowpox virus* and M*onkeypox virus* (MPXV) are capable of causing zoonotic infections in humans, and both species have a wide host range [9]. The orthopoxviruses are antigenically similar, and infection with one can induce long-lasting immunity against the others [10]. Only limited genomic data is available for orthopoxviruses endemic to North America, however they appear to be phylogenetically distinct from other orthopoxviruses [11].

The *Avipoxvirus* genus infects birds, can be transmitted via arthropod vectors as well as aerosols, and members are being investigated as vaccine vectors [12]. Although most chordopoxviruses share similar genome organization, many avipoxviruses contain an extensive genomic rearrangement relative to other ChPV [13]. Chordopoxviruses are often named for the animal from which they were isolated, although in many cases it is not the reservoir host. Ruminants such as cattle, goats, and sheep are the hosts of viruses in the *Capripoxvirus* genus [14]. Members of the *Cervidpoxvirus* genus are normally found in mule deer, but have recently been isolated from a gazelle, viruses of the *Leporipoxvirus* species infect rabbits, members of the *Suipoxvirus* species infect only swine, and primates are the hosts for members of the *Yatapoxvirus* genus [15,16].

Some members of ChPV are distinct from the others due to their high genomic GC content. The GC content in chordopoxviruses ranges from 25% in the capripoxviruses to 67% in squirrelpox virus [17], although a biological role for variation in GC content has not yet been identified. The high GC poxviruses include viruses in the *Molluscipoxvirus* genus, which is specific to humans, Nile crocodilepox virus, a virus in the *Crocodylidpoxvirus* genus, and members of the *Parapoxvirus* genus, which have been identified in hoofed mammals, shrews, weasels, and other members of the mammalian *Laurasiatheria* superorder [18,19,20,21].

The large host range of some poxviruses may be a result of host jumping, where a virus will occasionally infect an animal other than its typical host, and adaptive mutations may allow successful colonization of the new species [22,23]. DNA viruses have a comparatively low genetic mutation rate of approximately 10^−7^–10^−9^ mutations per site per round of replication which is closer to that of their hosts than to RNA viruses [24,25]. It is not well understood how the potentially large number of mutations necessary to cross host species barriers are able to accumulate quickly enough to allow the virus to adapt to a new host. Poxviruses are subject to several mechanisms which introduce genomic variability, including horizontal gene transfer (HGT), single nucleotide polymorphisms (SNPs), small insertions and deletions (indels), larger deletions of sequence which may include entire genes, and recombination [26]. The mechanisms utilized by viruses within any particular poxvirus genus to support their variation and evolution may differ from one genus to another. For example, the orthopoxviruses do not seem to have acquired any new genes through HGT since their most recent common ancestor, however, evidence supports progressive gene fragmentation and loss as one of the major determinants of speciation within the *Orthopoxvirus* genus [4]. Short indels are the most common cause for variation in orthopoxvirus gene content resulting in gene truncation, fragmentation, and loss [27].

Microsatellites, also called Simple Sequence Repeats (SSRs), or Short Tandem Repeats (STRs), are motifs of 1–6 nucleotides arranged in tandem repeats [28,29]. They are present in eukaryotes, prokaryotes, and viruses. The presence and characteristics of microsatellites have been shown to vary according to genome length and GC content, and the number and length of repeats varies by species and their location in coding and noncoding regions [30,31]. Microsatellites can function as hypervariable regions compared to surrounding non-repetitive genomic sequence. The motifs are not always replicated perfectly during DNA replication, which can lead to changes in the number of repetitions or single nucleotide polymorphisms (SNPs). Slipped-strand mispairing is one of the most likely causes of mutation in microsatellites, and occurs when DNA polymerase “slips” backwards or forwards along the template, and is facilitated by the short repeats [30,32]. When insertions and deletions (indels) occur in gene coding regions, they result in frameshift mutations and frequently therefore early stop mutations and truncation, unless the indel length is an in-frame multiple of 3, in which case the protein sequence will vary by the deletion or addition of an amino acid. Microsatellites can also serve as “hot spots” for recombination, which can lead to duplication or deletion of larger stretches of DNA, sometimes including whole genes [33,34].

Repetitive genetic elements have been shown to mediate different aspects of virus biology such as latency in some herpesviruses [35]. Poxviruses contain clusters of tandem repeats in their telomeres near the exterior ends of inverted terminal repeats bookending the genome, although the repeat units are longer than microsatellites [36]. The high variability often identified at short sequence repeats has led to great interest in characterizing microsatellites in many different species. Microsatellite distribution has been investigated in ssDNA viruses such as members of the family *Geminiviridae* that infect plants and the families *Circoviridae*, *Parvoviridae*, and *Anelloviridae* that infect vertebrates [37,38]. Short repeats were also identified throughout viruses in the *Herpesviridae* family, six genotypes of hepatitis C viruses, adenoviruses, influenza viruses, sin nombre virus, and human immunodeficiency virus type 1 [39,40,41,42,43].

Despite widespread identification of microsatellites and their functional significance in eukaryotic and prokaryotic genomes, the biological role of microsatellites in viral genomes remains largely unknown. We previously identified an inverse association between the length of orthopoxvirus genomes and the number of mutations they contain that could lead to truncated and fragmented genes [27]. While analyzing the data, we observed that many of the ESMs occurred at microsatellites (data was not reported). In this report, we have assessed the microsatellite content of viruses throughout the *Chordopoxvirinae* subfamily, characterized the microsatellites present in the viral genomes, and analyze the relationship between ESMs and microsatellites.

## 2. Materials and Methods

### 2.1. Genome Sequences and Assessment of Gene Content

We chose representative isolates from each genus within the *Chordopoxvirinae* subfamily, as well as each species within the Orthopoxvirus genus with available complete genome sequences (Table 1). Nucleotide sequences were downloaded from the Viral Bioinformatics Resource Center (vbrc.org) [44], with the exception of SQPV, which was downloaded from the National Center for Biotechnology Information (http://www.ncbi.nlm.nih.gov).

**Table 1:** Selected chordopoxvirus genomes used in these analyses.

Genus	Abbreviation	NCBI strain	Accession no.	Intact genes	Truncated genes	Fragmented genes	Length (bp)
*Species*							
Avipoxvirus							
*Fowlpox virus*	FWPV	Fowlpox virus strain Iowa	NC_002188	231	5	13	288,539
*Canarypox virus*	CNPV	Canarypox virus strain ATCC VR111	NC_005309	316	2	4	359,853
Capripoxvirus							
*Sheeppox virus*	SPPV	Sheeppox virus strain TU-V02127	NC_004002	150	1	8	149,955
*Goatpox virus*	GTPV	Goatpox virus strain Pellor	NC_004003	150	2	7	149,599
*Lumpy skin disease virus*	LSDV	Lumpy skin disease virus strain Neethling 2490	NC_003027	159	0	0	150,773
Cervidpoxvirus							
*Mule deerpox virus*	DPV	Deerpox virus W-848-83	NC_006966	172	0	1	166,259
Crocodylidpoxvirus							
*Nile crocodilepox virus*	CRV	Crocodilepox virus strain Zimbabwe	NC_008030	175	0	0	190,054
Leporipoxvirus							
*Myxoma virus*	MYXV	Myxoma virus strain Lausanne	NC_001132	166	0	1	161,773
*Rabbit fibroma virus*	RFV	Rabbit fibroma virus strain Kasza	NC_001266	161	0	4	159,857
Molluscipoxvirus							
*Molluscum contagiosum virus*	MOCV	Molluscum contagiosum virus strain subtype 1	NC_001731	159	0	0	190,289
Orthopoxvirus							
*Cowpox virus*	CPXV-Ger	Cowpox virus strain Germany 91-3	DQ437593	208	3	3	228,250
*Cowpox virus*	CPXV-BR	Cowpox virus strain Brighton Red	AF482758	206	3	1	224,499
*Cowpox virus*	CPXV-Gri	Cowpox virus strain GRI-90	X94355	209	3	0	223,666
*Vaccinia virus*	HSPV	Horsepox virus strain MNR-76	DQ792504	181	22	4	212,633
*Vaccinia virus*	RPXV	Rabbitpox virus strain Utrecht	AY484669	179	13	4	197,731
*Vaccinia virus*	VACV-WR	Vaccinia virus strain WR (Western Reserve)	AY243312	178	12	3	194,711
*Monkeypox virus*	MPXV-WR	Monkeypox virus strain MPXV-WRAIR7-61; Walter Reed 267	AY603973	175	7	14	199,195
*Monkeypox virus*	MPXV-ZAI	Monkeypox virus strain Zaire-96-I-16	AF380138	176	7	14	196,858
*Ectromelia virus*	ECTV	Ectromelia virus strain Moscow	AF012825	172	21	7	209,771
*Taterapox virus*	TATV	Taterapox virus strain Dahomey 1968	DQ437594	163	26	7	198,050
*Camelpox virus*	CMLV	Camelpox virus strain M-96 from Kazakhstan	AF438165	174	14	8	205,719
*Variola virus*	VARV-BRZ	Variola virus strain Brazil 1966 (v66-39 Sao Paulo)	DQ441419	162	18	12	188,062
*Variola virus*	VARV-SLE	Variola virus strain Sierra Leone 1969 (V68-258)	DQ441437	162	17	11	187,014
*Variola virus*	VARV-SAF	Variola virus strain South Africa 1965 (103 T'vaal, Nelspruit)	DQ441436	162	17	11	185,881
*Variola virus*	VARV-KWT	Variola virus strain Kuwait 1967 (K1629)	DQ441433	162	17	11	185,853
Parapoxvirus							
*Orf virus*	ORFV	Orf virus strain OV-SA00	NC_005336	134	0	0	139,962
*Bovine papular stomatitis virus*	BPSV	Bovine papular stomatitis virus strain BV-AR02	NC_005337	136	0	0	134,431
Suipoxvirus							
*Swinepox virus*	SWPV	Swinepox virus strain Nebraska 17077-99	NC_003389	151	0	0	146,454
Yatapoxvirus							
*Tanapox virus*	TANV	Tanapox virus strain Kenya	NC_009888	151	2	0	144,565
*Yaba monkey tumor virus*	YMTV	Yaba monkey tumor virus strain Amano	NC_005179	139	2	0	134,721
Unassigned							
Yoka poxvirus (not classified)	YKPV	Yoka poxvirus	NC_015960.1	164	12	5	175,699
*Squirrelpox virus*	SQPV	Squirrel poxvirus strain Red squirrel UK	NC_022563.1	142	0	0	148,803

As described in Hendrickson *et al*. [4] and Hatcher *et al*. [27], we used the Poxvirus Genome Annotation System (PGAS) to determine the coding potential of open reading frames (ORFs), to annotate predicted genes according to their coding state (*i.e*., intact, truncated, fragmented, or missing genes), and to compare syntenic regions in closely related strains or species. MAFFT alignments using the FFT-NS-2 method were generated for the avipoxviruses, the high GC isolates, and all isolates except the avipoxviruses, and were used to identify genes that have similar location but low homology [45]. An ORF was considered intact if it was similar in length to the longest ORF for all orthologs of that gene. Truncated genes were defined as genes greater than 30 amino acids long and retaining a predicted promoter sequence, but were less than 80% of the amino acid length of intact orthologs. This cutoff is based on a comparison of gene length conservation (described in Hendrickson *et al*. [4]). Fragmented genes were identified as genomic regions containing identifiable homologous sequence to an orthologous, intact ORF, but with the fragmented ORF coding for less than 30 amino acids, missing a predicted promoter region, or missing the 5' end of the gene, including a start codon.

ORFs that were flagged as potential genes through PGAS automated prediction but did not have an identified promoter, had an absent or weak Kozak consensus sequence, had a low Glimmer score, and showed no orthology with intact poxvirus genes were not labeled as genes or gene remnants unless they were identified as genes when the genome was originally published. To differentiate orthologous from paralogous genes, we assessed sequence homology as well as gene synteny, the conservation of genomic location and gene neighbors [46]. We utilized the term “syntelog” to represent orthologous genes shared across isolates with a common syntenic genome location [4].

### 2.2. Detection of Early Stop Mutations (ESMs)

ESMs are defined as mutations that give rise to a stop codon in the sequence of a gene that either interrupts the start codon or truncates the ORF to a length 80% or less of the length of the intact orthologous gene. ESMs were identified as in Hatcher *et al*. [27] by visualizing the nucleotide sequence of each open reading frame and the corresponding amino acid translation of the coding frame and the two alternate frames, and annotating mutations that introduced either a stop codon in the normal coding frame, introduced an indel resulting in a frameshift mutation and subsequent early stop codon in the new reading frame, or altered the start codon of a gene. In the case of an altered reading frame, the mutation that caused the change in reading frame is coded as the ESM and not the newly introduced (now in-frame) stop codon.

### 2.3. Microsatellite Identification

Each of the viruses was assessed individually for the presence of microsatellites using the IMEx program available at http://imex.cdfd.org.in [42]. Repeats with motifs of 1–6 bp were identified if present with a minimum repeat number of 4 for motifs of 1 base, 3 for motifs of 2 or 3 bases, and 2 for motifs of 4, 5, or 6 bases. Perfect and imperfect repeats were allowed, and the imperfections were limited to a variation of 10% within the repeat tract. Microsatellite content was determined by dividing the total number of nucleotides present within the repeats by the length of the genome. When counting ESMs that overlapped microsatellites, if an ESM only partially overlapped a microsatellite, it was not included in the number of ESMs overlapping repeats.

### 2.4. Statistical Analysis

The total number of microsatellites was normalized according to genome size. Relative abundance was calculated as the number of microsatellites per kilobase of genomic sequence. Relative density was calculated as the sum total of microsatellite nucleotides per kilobase of genomic sequence.

Linear regression was used to determine the relationship between various genomic features and microsatellites, and was performed in Microsoft Excel 2010.

## 3. Results

### 3.1. Assessment of Chordopoxvirus Gene Content

**Figure 1:**
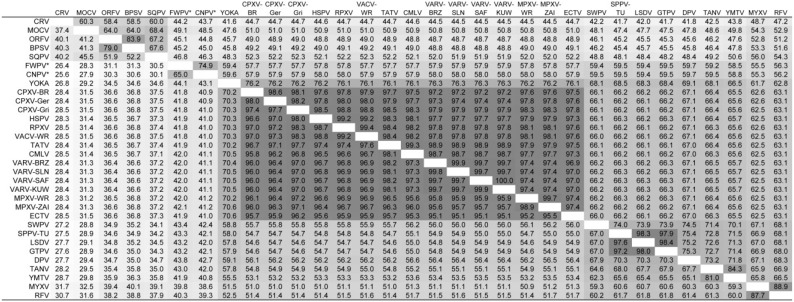
Pairwise nucleotide % identities for the conserved central region of the poxvirus isolates used in this study. Values below the diagonal included gaps when calculating the pairwise identity, whereas values above the diagonal had all gapped columns removed before analysis. The conserved central region spans the genomic sequences from Cop-F9L (CPXV-Ger position 52,398) to Cop-A34R (CPXV-Ger position 160,094). Darker shading reflects higher levels of identity. *FWPV and CNPV, contain a genome inversion in this region, and therefore their sequences were rearranged in order to align them with the other viruses.

We annotated the genes of representative viruses from each chordopoxvirus genus, and characterized those genes as intact, truncated, or fragmented (Table 1). Using whole genome multiple sequence alignments, we were able to use homology and synteny to assist with gene annotation, including identifying truncated and fragmented genes by comparing the genomic sequence in these regions with the sequences of intact, syntenic genes present in other isolates. When identifying non-intact genes, we define truncated genes as less than 80% the length of intact genes but with the 5' end intact, as opposed to fragmented genes which maintain nucleotide sequence homology to intact genes but either do not have an intact 5' end of the gene, or the remaining orthologous ORF is less than 30 amino acids long. We were able to identify at least one non-intact gene in most of the viruses, although the majority were in orthopoxviruses (reported in Hatcher *et al*. [27]), yoka poxvirus, capripoxviruses, and the avipoxviruses. Yoka poxvirus is the closest phylogenetic relative to the orthopoxviruses which has been identified at this time [3]. We were unable to identify any truncated or fragmented genes in the high GC viruses. Figure 1 provides a comparison of the similarities of the isolates as pairwise nucleotide identities for the most conserved central portion of the genomes.

### 3.2. Assessment of Truncated and Fragmented Genes, and Early Stop Mutation Content

Genome reduction through gene fragmentation and loss is one of the primary drivers of speciation in the orthopoxviruses, and previously we described an analysis of early stop mutations (ESMs) leading to gene truncation and fragmentation in orthopoxviruses [27]. We expanded that analysis here, characterizing the ESMs in viruses in the other genera of the *Chordopoxvirinae* subfamily, and were able to identify the causative ESMs for all but two of the non-intact genes (Table 2). The two instances were due to high variability between the non-intact genes and their homologs, which prevented us from being able to reliably identify the ESMs.

Orthopoxvirus genes range from highly intact in cowpox viruses (0.013–0.026 non-intact genes per 1000 bp) to highly fragmented in the variola viruses and TATV (0.150–0.167 non-intact genes per 1000 bp). Orthopoxvirus truncated and fragmented genes are interrupted by an average of 2.8 ESMs per gene, similar to the average for the entire subfamily, although some isolates average as high as five ESMs per non-intact gene. YKPV is not only genetically similar to the orthopoxviruses, but has a comparable number of truncated or fragmented genes (17 in YKPV and an average of 20.7 in orthopoxviruses), and ESMs per nonintact gene (2.8 in both YKPV and orthopoxviruses).

**Table 2:** Comparative genomic content of early stop mutationss and microsatellites.

Virus Genome	ESM Count	Non-intact genes	Non-intact genes/1000 bp	ESMs/non-intact gene	ESMs overlapping microsatellites	% of ESMs overlapping microsatellites	Genome microsatellite content (%)	GC% of genome	GC% of microsatellites
FWPV	53	18	0.062	2.9	15	28.3	24.0	31	15
CNPV	22	6	0.017	3.7	9	40.9	23.5	30	22
SPPV	107	9	0.060	11.8	66	61.7	31.7	25	9
GTPV	82	9	0.060	9.1	56	68.3	31.7	25	10
LSDV	0	0	0	-	0	-	31.1	26	10
DPV	2	1	0.006	0.0	1	50.0	29.7	26	10
CRV	0	0	0	-	0	-	22.2	62	66
MYXV	2	1	0.006	1.0	1	50.0	20.8	44	35
RFV	44	4	0.025	11.0	11	25.0	21.1	40	28
MOCV	0	0	0	-	0	-	22.5	63	70
CPXV-Ger	11	6	0.026	1.8	7	63.6	22.5	34	20
CPXV-BR	3	4	0.018	0.8	1	33.3	22.9	33	20
CPXV-Gri	2	3	0.013	0.7	1	50.0	22.5	34	20
HSPV	49	26	0.122	1.9	29	59.2	22.6	33	19
RPXV	44	17	0.086	2.6	36	81.8	22.5	34	19
VACV-WR	41	15	0.077	2.7	31	75.6	22.8	33	19
MPXV-WR	104	21	0.105	5.0	66	63.5	22.7	33	19
MPXV-ZAI	95	21	0.107	4.5	56	58.9	22.6	33	19
ECTV	73	28	0.133	2.6	37	50.7	22.5	33	19
TATV	87	33	0.167	2.6	55	63.2	22.7	33	19
CMLV	65	22	0.107	3.0	37	56.9	22.7	33	19
VARV-BRZ	92	30	0.160	3.1	60	65.2	22.6	33	18
VARV-SLE	100	28	0.150	3.6	67	67.0	22.6	33	18
VARV-SAF	93	28	0.151	3.3	58	62.4	22.6	33	18
VARV-KWT	93	28	0.151	3.3	59	63.4	22.7	33	18
ORFV	0	0	0	-	0	-	22.8	64	68
BPSV	0	0	0	-	0	-	12.2	65	76
SWPV	0	0	0	-	0	-	27.1	28	12
TANV	4	2	0.014	1.0	1	25.0	30.2	27	10
YMTV	4	2	0.015	1.5	3	75.0	28.0	30	12
YKPV	47	17	0.097	2.8	21	44.7	28.8 *	26	11
SQPV	0	0	0	-	0	0.0	23.5	67	73
Total	1140	379	0.060*	3.4 *	784	68.8 *	24.1	37 *	26 *

* These figures are averages, not totals.

In the avipoxviruses, FWPV has many more truncated and fragmented genes than CNPV (18 *versus* 6), a greater number of ESMs (53 *versus* 22), but the frequency of ESMs per nonintact gene in FWPV is lower than in CNPV (2.9 *versus* 3.7). Two members of the capripoxviruses have nine non-intact genes each, with an average of 11.8 ESMs per gene in SPPV, and an average of 9.1 in GTPV. The third capripoxvirus isolate included, LSDV, does not appear to have any truncated or fragmented genes. Because we were unable to identify truncated or fragmented genes in the high GC content viruses, we were also unable to identify ESMs in those viruses. It is difficult to say whether the lack of non-intact genes may be due to the low number of sequenced isolates available for high GC viruses compared to high AT viruses, or due to different evolutionary mechanisms that prevent the accumulation of ESMs. The availability of additional sequenced high GC poxvirus isolates may provide the data necessary to answer this question.

**Figure 2:**
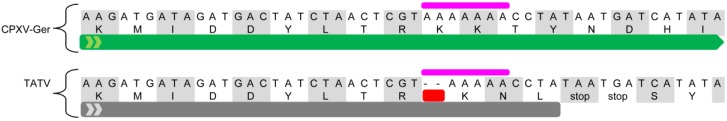
Example of an early stop mutation (ESM) overlapping a microsatellite. For each isolate, a portion of the coding sequence for the thymidylate kinase gene (Cop-A48R) is shown. The top row is the nucleotide sequence, the amino acid translation is the middle row, and a bar is shown to indicate the gene ORF. The green bar represents the intact gene in CPXV-Ger which continues off the right of the figure, and the grey bar represents the homologous truncated gene in TATV. The pink bars highlight microsatellites consisting of a monomeric repeat. An ESM in TATV in the form of a 2 bp deletion is shown by the red bar.

**Figure 3:**
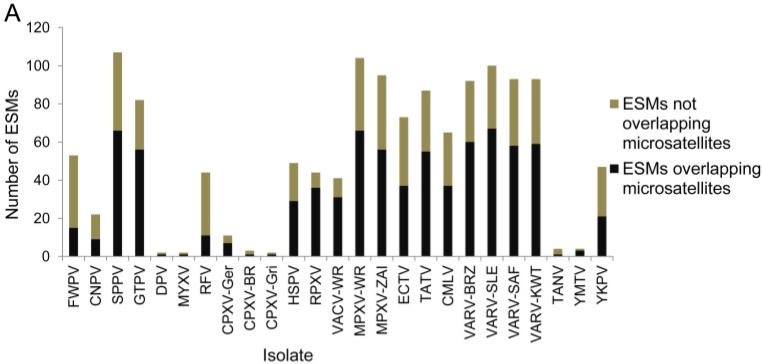

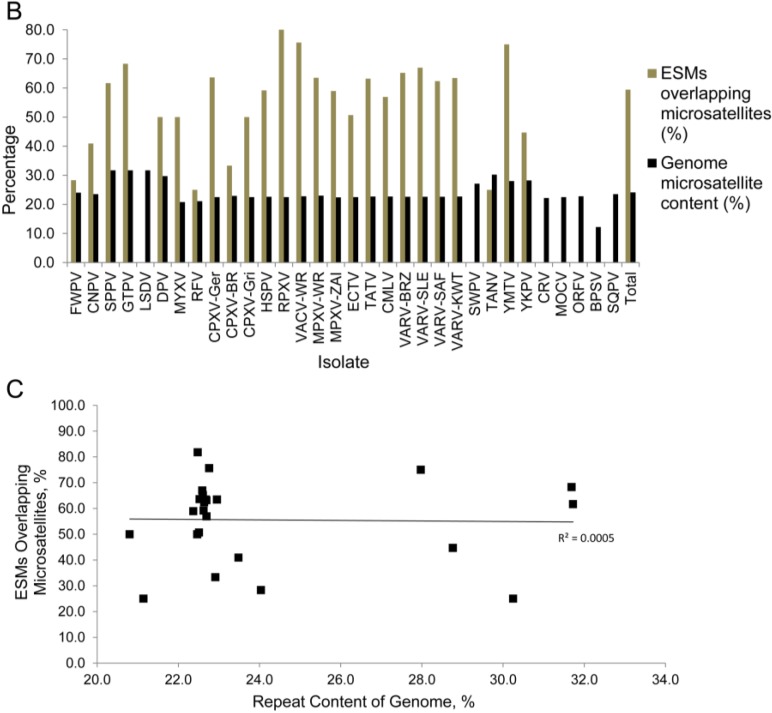
Presence of early stop mutations at microsatellites is independent of repeat content of the genome. (**a**) The number of ESMs which overlap or do not overlap microsatellites. Only isolates with non-intact genes and ESMs are shown; (**b**) The percentage of ESMs which overlap microsatellites is shown, along with the percentage of each genome which consists of microsatellites; (**c**) No relationship could be identified between the percentages of ESMs which overlap microsatellites and the percentage of each genome which consists of microsatellites.

Short repeats in DNA sequences are associated with higher rates of mutation in many species, and we wanted to understand if microsatellites may have a role in the introduction of early stop mutations in poxviruses. To determine if there is an association between ESMs and short repeats, we identified the microsatellites in the genomes, and identified the ESMs that occurred at the microsatellites (Figure 2). The short repeats are present in all of the isolates and account for an average 24% of the genome in chordopoxviruses, ranging from 12.2% in BPSV to 31.7% in the capripoxviruses (Table 2). The orthopoxviruses are within a very narrow range of 22.4%–23.0% of the genome consisting of repeats. In most viruses where ESMs were identified, a greater number of ESMs are located at microsatellites than can be explained by the microsatellite content of the genomes (Table 2 and Figure 3A,B). In some examples, the incidence of colocalization is striking, for example in RPXV, 81.1% of the ESMs occur at microsatellites, but the microsatellites comprise only 22.5% of the genome. This is not a universal observation however since in FWPV, RFV, and TANV, the distributions of ESMs and the genome microsatellite content are close to equal. We did not, however, find any correlation between the frequency of ESMs overlapping microsatellites, and the overall genome microsatellite content (Figure 3C).

### 3.3. Chordopoxvirus Microsatellite Content

**Figure 4:**
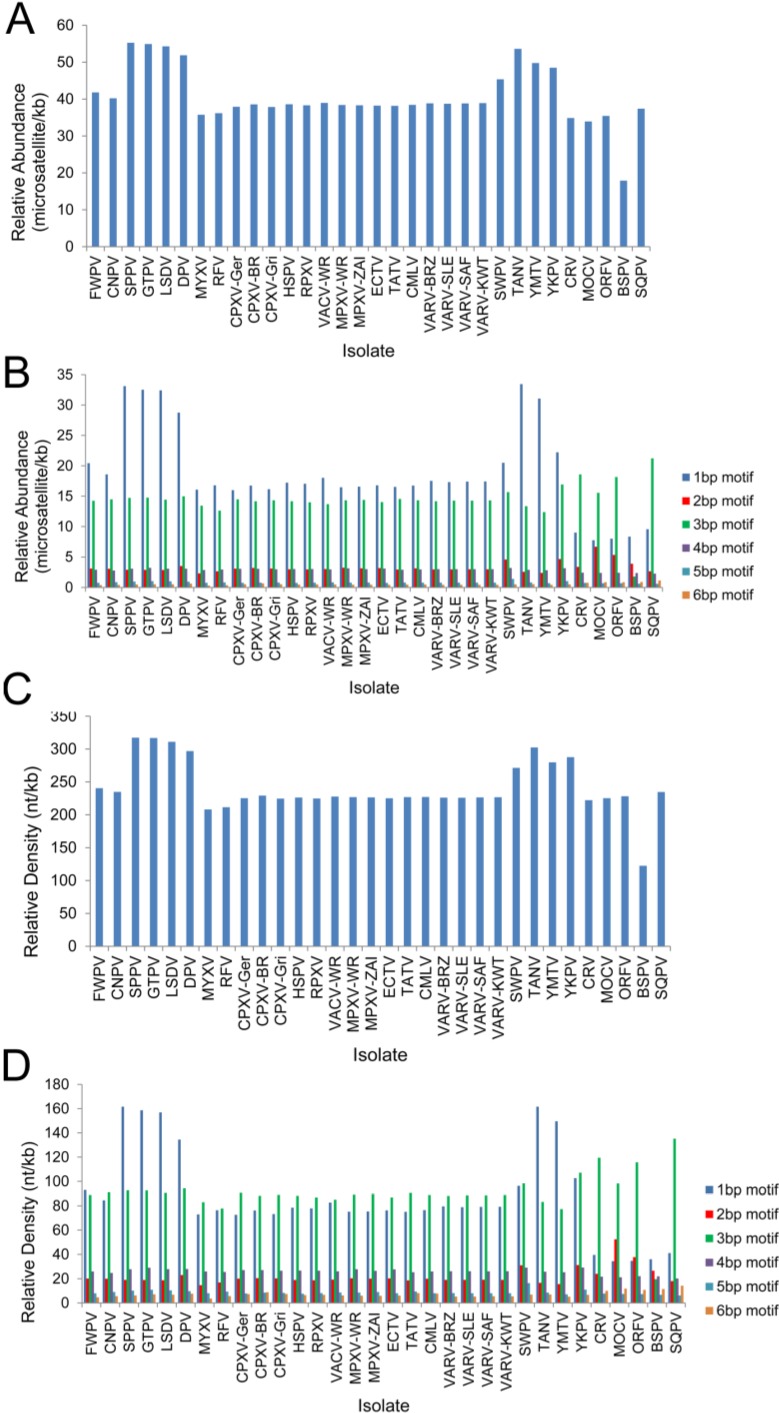
Relative abundance and density of microsatellites. RA for (**a**) all microsatellites and for (**b**) each motif length measured (1 bp to 6 bp) for each isolate. RD for (**c**) all microsatellites and for (**d**) each motif length measured for each isolate. The high GC viruses are shown at the right end of each figure.

**Figure 5:**
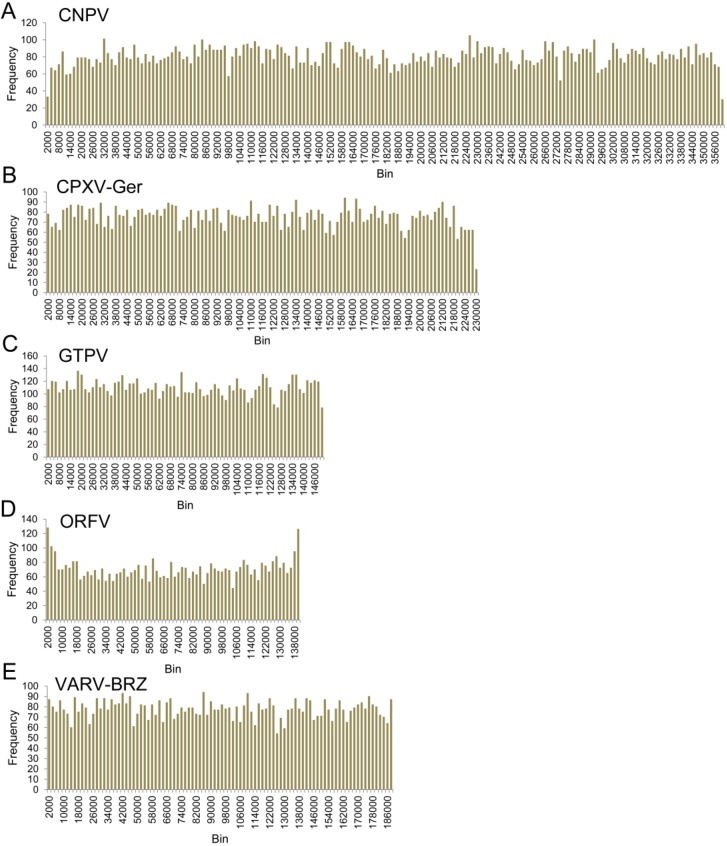
Distribution of microsatellites across genomes. The number of microsatellites is shown for bins of 2000 bp. The number of bins reflects the length of each genome. The isolates shown are (**a**) CNPV; (**b**) CPXV-Ger; (**c**) GTPV; (**d**) ORFV; and (**e**) VARV-BRZ.

Counts of microsatellites of oligonucleotides from 1–6 bp motifs are described in Table 3 and Figure 4. The relative abundance (RA) of microsatellites, or the average number of microsatellites in every kilobase of genomic sequence varies from 18 in BSPV to 55 in SPPV and GTPV. The orthopoxviruses are very similar to each other, and each member has a RA between 34 and 39 microsatellites/kb. As the length of repeat motifs increases, the number of repeats decreases. Although the reported hexamer RA for several of the viruses is close to 0, repeats of 6 bp motifs were observed in all of the viruses. The portion of the genome made up of microsatellites is the relative density (RD), and ranges from 122 microsatellite nucleotides per kilobase of genomic nucleotides in BSPV to 317 nt/kb in SPPV and GTPV. The most numerous repeats are monomers and trimers, with the greatest number of repeats being monomers in high AT viruses, with high GC viruses having greater numbers of trimers. This is true whether comparing actual counts or RA for each virus, however because RD takes into account the size of each repeat, there is no clear pattern. Microsatellites appear to be distributed relatively evenly across genomes, as shown in Figure 5; however, there is a significant difference between the RA of microsatellites in noncoding and coding regions (T test, *p* < 0.0001, 95% CI of the difference between the means is 10.146–26.034). The mean RA in noncoding regions is 57 microsatellites/1000 bp, and in coding regions is 39 microsatellites/1000 bp. This is best explained by more relaxed selection pressure on noncoding regions, where mutations due to the increased variability in microsatellites are less likely to affect virus viability.

**Table 3:** Description of the microsatellite sequences identified in *Poxviridae* genomes. Comparative abundance of repeats is shown. The high GC viruses are shown at the bottom of the table.

Isolate	Total number	Total number of 1, 2, 3, 4, 5, 6 bp microsatellites	RA	RA of 1, 2, 3, 4, 5, 6 bp Microsatellites *^a^*	Total length of microsatellites	RD	RD of 1, 2, 3, 4, 5, 6 bp microsatellites	RA in noncoding regions	RA in coding regions
FWPV	12063	5896, 894, 4110, 838, 217, 108	42	20, 3, 14, 3, 1, 0^a^	69359	240	93, 20, 89, 26, 8, 5	49	40
CNPV	14465	6687, 1100, 5214, 999, 310, 147	40	19, 3, 14, 3, 1, 0^a^	84508	235	85, 20, 91, 25, 9, 5	47	39
SPPV	8283	4964, 435, 2207, 461, 143, 73	55	33, 3, 15, 3, 1, 0^a^	47574	317	162, 19, 93, 28, 10, 6	85	53
GTPV	8214	4863, 431, 2209, 481, 154, 76	55	33, 3, 15, 3, 1, 1	47406	317	159, 19, 92, 29, 11, 7	91	52
LSDV	8181	4888, 430, 2173, 463, 149, 78	54	32, 3, 14, 3, 1, 1	46886	311	157, 19, 91, 28, 10, 7	129	51
DPV	8620	4780, 583, 2490, 510, 154, 103	52	29, 4, 15, 3, 1, 1	49369	297	134, 23, 94, 28, 10, 8	81	49
MYXV	5780	2599, 373, 2174, 464, 123, 47	36	16, 2, 13, 3, 1, 0^a^	33652	208	73, 15, 83, 26, 8, 4	88	33
RFV	5781	2683, 423, 2018, 469, 130, 58	36	17, 3, 13, 3, 1, 0^a^	33800	211	76, 17, 78, 25, 9, 6	62	34
CPXV-Ger	8648	3650, 704, 3307, 698, 173, 116	38	16, 3, 14, 3, 1, 1	51415	225	73, 20, 91, 27, 8, 7	62	35
CPXV-BR	8656	3758, 716, 3178, 685, 172, 147	39	17, 3, 14, 3, 1, 1	51443	229	76, 20, 88, 27, 8, 9	57	36
CPXV-Gri	8466	3613, 691, 3202, 668, 178, 114	38	16, 3, 14, 3, 1, 1	50251	225	73, 20, 89, 27, 8, 7	53	36
HSPV	8205	3662, 632, 3012, 643, 158, 98	39	17, 3, 14, 3, 1, 0^a^	48109	226	79, 19, 88, 27, 8, 6	45	37
RPXV	7570	3370, 584, 2766, 596, 154, 100	38	17, 3, 14, 3, 1, 1	44447	225	78, 19, 87, 27, 8, 7	44	37
VACV-WR	7586	3506, 588, 2664, 574, 162, 92	39	18, 3, 14, 3, 1, 0^a^	44323	228	83, 19, 85, 26, 9, 6	45	37
MPXV-WR	7645	3281, 643, 2854, 626, 155, 86	38	16, 3, 14, 3, 1, 0^a^	45187	227	75, 20, 89, 28, 8, 6	44	37
MPXV-ZAI	7540	3262, 617, 2830, 589, 157, 85	38	17, 3, 14, 3, 1, 0^a^	44559	226	75, 20, 90, 26, 9, 6	46	37
ECTV	8018	3519, 667, 2942, 651, 153, 86	38	17, 3, 14, 3, 1, 0^a^	47220	225	76, 20, 87, 28, 8, 6	45	36
TATV	7559	3273, 582, 2880, 575, 157, 92	38	17, 3, 15, 3, 1, 0^a^	44943	227	75, 19, 91, 25, 9, 8	42	37
CMLV	7900	3446, 644, 2945, 607, 159, 99	38	17, 3, 14, 3, 1, 0^a^	46688	227	77, 20, 89, 26, 8, 8	44	37
VARV-BRZ	7305	3294, 558, 2665, 564, 146, 78	39	18, 3, 14, 3, 1, 0^a^	42513	226	80, 19, 88, 26, 8, 5	43	38
VARV-SLE	7241	3237, 554, 2667, 561, 145, 77	39	17, 3, 14, 3, 1, 0^a^	42251	226	79, 19, 89, 26, 8, 5	43	37
VARV-SAF	7214	3234, 553, 2650, 556, 143, 78	39	17, 3, 14, 3, 1, 0^a^	42078	226	79, 19, 89, 26, 8, 5	43	37
VARV-KWT	7228	3238, 553, 2658, 557, 144, 78	39	17, 3, 14, 3, 1, 0^a^	42140	227	79, 19, 89, 26, 8, 5	43	38
SWPV	6642	3001, 672, 2296, 467, 205, 74	45	20, 5, 16, 3, 1, 1	39737	271	96, 31, 98, 29, 16, 7	68	44
TANV	7746	4835, 371, 1929, 415, 117, 79	54	33, 3, 13, 3, 1, 1	43730	302	162, 17, 83, 26, 8, 7	67	52
YMTV	6703	4184, 322, 1668, 379, 93, 57	50	31, 2, 12, 3, 1, 0^a^	37689	280	150, 15, 77, 25, 7, 5	63	48
YKPV	8517	3902, 821, 2973, 554, 180, 87	48	22, 5, 17, 3, 1, 0^a^	50540	288	103, 31, 107, 29, 11, 7	60	47
CRV	6626	1711, 640, 3533, 460, 139, 145	35	9, 3, 19, 2, 1, 1	42209	222	40, 24, 119, 22, 8, 10	62	34
MOCV	6456	1475, 1274, 2960, 454, 130, 163	34	8, 7, 16, 2, 1, 1	42852	225	34, 52, 98, 21, 7, 12	41	32
ORFV	4959	1123, 748, 2540, 335, 94, 119	35	8, 5, 18, 2, 1, 1	31940	228	35, 38, 116, 22, 7, 11	30	36
BSPV	2404	1123, 522, 240, 318, 80, 121	18	8, 4, 2, 2, 1, 1	16437	122	36, 27, 20, 22, 7, 11	34	17
SQPV	5565	1423, 393, 3159, 337, 85, 168	37	10, 3, 21, 2, 1, 1	34911	235	41, 18, 135, 20, 6, 14	56	36

*^a^* The RA of 6bp microsatelites is closer to 0 than to 1, but is not equal to 0.

**Figure 6:**
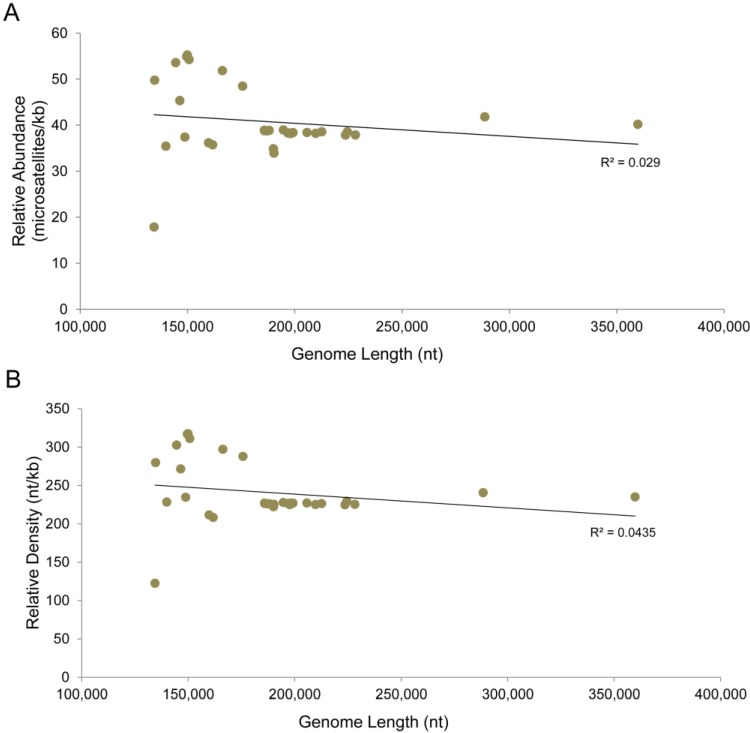
Genome length is not correlated with microsatellite abundance or density. (**a**) RA and (**b**) RD are shown for each isolate, plotted *versus* genomic length.

**Figure 7:**
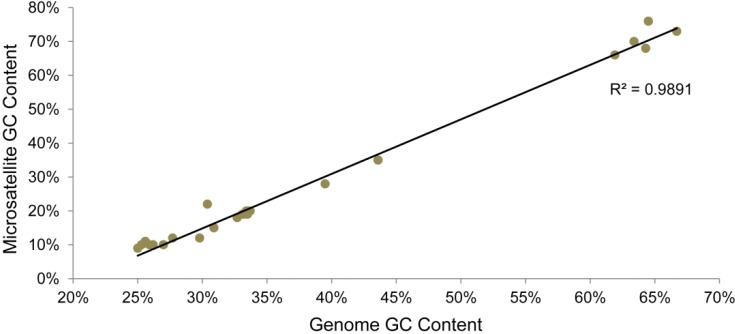
Correlation of microsatellite GC content and genome GC content.

We next attempted to identify genomic characteristics that may be associated with microsatellite content. The genome length for viruses included in this study ranges from approximately 134,000–360,000 bp. Neither RA nor RD show significant correlation with genome size in the chordopoxvirus isolates we analyzed (Figure 6). We also analyzed the microsatellites for their GC content, since it has previously been shown that the GC content of the genome is associated with the microsatellite composition in other organisms [31,47]. All of the microsatellites in a virus were assessed for the proportion of G and C nucleotides they contained, and this number was compared to the total GC content of each genome. In these poxviruses, the GC content of the microsatellites was strongly associated with the GC content of the genome (Figure 7).

## 4. Discussion

Poxviruses have been shown to evolve through a combination of vertical gene transfer, horizontal gene transfer from eukaryotes and other viruses, genetic drift, and, at least in the orthopoxviruses, gene loss. In this report, we cataloged all of the truncated and fragmented genes present in completely sequenced genomes of representative poxviruses from the *Chordopoxvirinae* subfamily, and we identified the early stop mutations leading to these gene truncations and fragmentations. We also showed that these ESMs were associated with microsatellites, and we characterized the microsatellite content in the genomes.

We observed a wide variation in the number of nonintact genes both in the subfamily as a whole, and often within genera. While reductive evolution has commonly been accepted as a major form of adaptation in the orthopoxviruses, this finding may signify that it is also active in other poxvirus genera such as the avipoxviruses. Although FWPV and CNPV share a similar genomic arrangement, CNPV contains over 75 kbp of additional sequence compared to FWPV [48]. FWPV also contains three times as many nonintact genes compared to CNPV (Table 2). Therefore, genome reduction through a process of gene truncation and fragmentation may have played a significant role in the evolution of FWPV. Similar features can be seen in the genus *Capripoxvirus*, where LSDV appears to only code for intact genes. In comparison, SPPV and GTPV each have nine non-intact genes.

One of the limitations with this study is that gene truncations and fragments cannot be identified without an example of an intact gene. When analyzing genomes for genes that are truncated or fragmented, we were unable to identify any in the high GC viruses. There are at least two possible interpretations for this. The high GC viruses contain a large number of unique genes and a low number of viruses sequenced for each genus, making it difficult to identify a gene as truncated or fragmented since there is no intact gene in a closely related virus with which to compare. Because of this, several genes may be classified as intact, when they are in fact truncated or fragmented. The second possible reason is that the high GC viruses may be subject to different evolutionary mechanisms, including a lack of gene reduction as compared to viruses with higher AT content and higher numbers of nonintact genes. However, a number of non-high GC viruses also contain low numbers of nonintact genes including LSDV, DPV, SWPV, the leporipoxviruses, and the yatapoxviruses. In addition, CPXV isolates only contain 3–6 nonintact genes in contrast to the other orthopoxviruses that contain between 15 and 33 nonintact genes. This emphasizes that GC content alone does not directly determine the number of nonintact genes present in any single genome, and that other factors must play a role in determining the extent to which gene truncation and fragmentation play a role in the evolution of poxvirus species.

While most viruses show an association between ESMs and microsatellites, this does not appear to be the case for FWPV, RFV, and TANV, (Table 2, Figure 3B). This may be due to different mechanisms being used for the introduction of ESMs compared to other poxviruses, but it may also be due to the accumulation of mutations in microsatellites that originally colocalized with ESMs, but whose sequence has now diverged significantly making it difficult to identify the former microsatellites.

Another interesting observation is the relatively low number of microsatellites in BPSV. BPSV and ORFV are both in the genus *Parapoxvirus*, and have genomes that are 134,431 and 139,952 bp long, respectively. We were unable to identify any non-intact genes in either ORFV or BPSV (both are high GC viruses). BPSV has a genomic microsatellite content of 12.2%, compared to ORFV, which is 22.8%, and 20.8%–31.7% for all other chordopoxviruses.

DNA viruses, and especially dsDNA viruses, have been shown to have relatively low mutation rates, more similar to eukaryotes than to RNA viruses [25]. Therefore, mechanisms that would support an increase in the number of mutations or that would target mutations to specific regions of the genome may provide an opportunity to adapt to alterations in the environment, selecting for new genome variants that increase the fitness of the virus. Recently, Elde *et al*. identified just such a mechanism for vaccinia virus that was able to expand the copy number of an immunomodulatory gene when exposed to selective conditions favoring expression of that gene [49]. The genomic regions containing the gene were most likely duplicated by recombination, however the authors also witnessed loss of the duplications when selection pressure was removed. Gene duplication and reduction may be one method of rapidly altering the poxvirus genome. Increased numbers of mutations due to the presence of microsatellites may be another. The two methods are not mutually exclusive, since non-homologous recombination preferentially occurs at repeat regions [33,34], and this may be a mechanism by which the accordion-like expansion and contraction of genes like the instance observed by Elde *et al*. may occur.

## 5. Conclusions

This study highlights the role of microsatellites in poxvirus sequence diversity, and how they may affect viral host range and pathogenesis by playing a role in gene variation and inactivation. We found a high incidence of statistically significant co-localization of ESMs with microsatellites. Genes which are no longer impacted by selection pressure to remain functional may be more likely to accumulate mutations at microsatellites, since these short repeats often show higher variability than the surrounding sequences [50,51]. Microsatellites may therefore serve as a major source of genomic variability in chordopoxviruses, contributing to the introduction of ESMs and therefore impacting gene content, virus biology, and evolution. Microsatellite hypervariability may allow the virus to adapt more quickly to environmental changes, such as the phenotypic changes needed when adapting to newly encountered hosts.
